# Sociodemographic and Psychological Factors Influencing Human Milk Donation: A Cross-Sectional Study of 420 Lactating Mothers in Vijayapura

**DOI:** 10.7759/cureus.71963

**Published:** 2024-10-20

**Authors:** Vasudha Bodi Jayakrishna, Shankargouda V Patil, Mallanagouda Patil

**Affiliations:** 1 Pediatrics, Shri BM Patil Medical College Hospital and Research Centre, BLDE(Deemed to be University), Vijayapura, IND

**Keywords:** breastfeeding, human milk banking, milk donation, neonatal health, public health, socio-demographic factors

## Abstract

Background

Breastfeeding is crucial for infant health, but not all mothers can breastfeed due to various constraints. Making human milk banks (HMBs) is essential for providing donor milk to preterm and sick infants.

Methods

This is a cross-sectional study at Shri BM Patil Medical College Hospital and Research Centre in Vijayapura involving 420 lactating mothers. Convenience sampling and semi-standardized interviews were used to assess socio-demographics, maternal factors, and attitudes toward milk donation.

Results

Among the 420 lactating mothers surveyed, urban mothers were significantly more likely to have received breastfeeding counseling (56.7% vs. 41.4%) and were more aware of milk bank donations (20.0% vs. 11.4%) than rural mothers. Logistic regression analysis revealed that urban residence, higher education, and prior knowledge of milk donation were significant predictors of willingness to donate breast milk.

Conclusion

The findings emphasize the importance of education and awareness, particularly in rural areas, to promote milk donation. Addressing socio-economic disparities and sharing personal experiences can also encourage donations. Targeted educational programs are crucial for enhancing donation rates and improving newborn health outcomes, making milk donation a vital area for public health initiatives.

## Introduction

Breastfeeding is the best way to provide critical nutrients for healthy newborn growth and development [1]. It provides comprehensive nutrition and immune protection and promotes physical and cognitive development [2,3]. Due to medical, societal, and economic constraints, not all mothers can breastfeed despite its many advantages. Human milk banking helps infants who cannot breastfeed obtain nutritional and protective benefits. Human milk banks (HMBs) collect, filter, process, and distribute donor milk, saving preterm and sick infants [4,5].

Breastfeeding has several health benefits for mothers and newborns beyond simple nourishment. Breast milk provides important fatty acids, lactose, vitamins, and minerals for brain and body development. Its antibodies, lymphocytes, and other bioactive components dramatically lower the incidence of respiratory infections, gastrointestinal problems, and other pediatric ailments [6-9]. Breastfeeding improves sensory and cognitive development, boosting intelligence quotient (IQ) and school achievement. Despite these benefits, conditions like mastitis, HIV, and lactation insufficiency, frequently caused by hormone imbalances or breast operations, can make breastfeeding difficult. Socioeconomic variables, including returning to work, lack of family support, and inadequate maternity leave legislation, make exclusive breastfeeding harder [10-12]. These issues emphasize the necessity for human milk banks to meet babies' nutritional and developmental needs when direct breastfeeding is impossible. The voluntary donation of extra breast milk to human milk banks for newborns who cannot be nursed is a vital practice. This procedure is crucial because it guarantees that fragile newborns, such as preterm, low-birth-weight, or medically compromised ones, receive the best nutrition and immunological protection from human milk.

Human milk provided to these newborns can reduce the risk of necrotizing enterocolitis, sepsis, and other serious diseases [13-16]. HMBs gather, filter, process, and distribute donated milk. Donors are carefully screened to ensure milk safety and quality. After collection, milk is pasteurized to kill germs and kept under regulated conditions until use. This systematic strategy preserves donor milk's nutritional and immunological characteristics, saving newborns.

Human milk donation in India has untapped potential and cultural barriers [17]. Regional culture strongly influences milk donation, with many women perceiving breast milk as a personal and familial resource rather than a community one [18-20]. This notion may discourage donation. Educational background and awareness also shape mothers' milk donation views. Mothers with higher education and understanding of breast milk advantages are more likely to donate [21]. 

Despite the well-documented benefits of breastfeeding, socio-demographic and psychological barriers continue to hinder human milk donation in Vijayapura. Enhanced milk bank protocols and increased availability of human milk could significantly decrease neonatal mortality and morbidity for vulnerable newborns. This study aims to inform targeted educational interventions to increase donation rates, thereby improving neonatal health outcomes in both urban and rural areas. Further research and initiatives will promote milk donation and enhance the health and development of newborns who need it most. 

## Materials and methods

This cross-sectional study aimed to explore factors influencing breast milk donation willingness among lactating mothers. Convenience sampling was employed to recruit 420 lactating mothers from the postnatal ward of Shri BM Patil Medical College Hospital and Research Centre, Vijayapura. Institutional Ethical Clearance was taken from BLDE (Deemed to be University) with the approval number BLDE(DU)/IEC/651/2022-23. 

Data were collected through semi-standardized interviews conducted by trained research assistants, and statistical analyses, including chi-square tests and logistic regression, were performed using SPSS Version 20. Participants were selected based on inclusion criteria, including hospitalization at the medical college, excluding those with certain health conditions like sexually transmitted diseases (STDs), COVID-19, psychiatric disorders, and anti-epileptic medication use, ensuring participant safety and data integrity. Inferential analyses included independent t-tests and chi-square tests to compare urban and rural groups on continuous and categorical variables. Logistic regression identified socio-demographic and knowledge factors associated with donation willingness, reporting crude and adjusted odds ratios with 95% CIs.

## Results

The study population comprised 420 lactating mothers, equally divided between urban (Group 1) and rural (Group 2) settings. The difference in age group distribution and educational status between urban and rural participants was statistically significant, with a chi-square value of 5.6334 (p-value 0.018) and a chi-square value of 96.9285 (p-value of 0.000), respectively. Urban mothers were significantly more likely to have received breastfeeding counselling during antenatal visits (56.7% vs 41.4%), to be currently breastfeeding (83.8% vs 73.8%), and to be aware of milk bank donation (20.0% vs 11.4%, p=0.016). History of NICU admission for a previous baby (AOR 8.46, 95% CI:2.84-12.46, p<0.001) was significantly associated with an increased willingness to donate breast milk.

The age distribution revealed that 52.4% of urban mothers and 63.8% of rural mothers were aged 18-25 years, while 47.6% of urban and 36.2% were aged 26-36 years. The difference in age group distribution between urban and rural participants was statistically significant, with a chi-square value of 5.6334 and a p-value of 0.018 (Table 1). 

**Table 1:** Distribution of age groups based on the location of the study participants.

Age in years	Urban n (%)	Rural n (%)
18 - 25	110 (52.4)	134 (63.8)
26 - 36	100 (47.6)	76 (36.2)
Total	210 (100)	210 (100)

In the rural group, 7.1% (n=15) were illiterate, 60.5% (n=127) had education ranging from class 1 to 12, 31.0% (n=65) had a bachelor's degree, and 1.4% (n=3) had a master's degree. In the urban group, 1.9% (n=4) were illiterate, 18.1% (n=38) had education ranging from class 1 to 12, 75.2% (n=158) had a bachelor's degree, and 4.8% (n=10) had a master's degree. The difference in educational status between urban and rural participants was statistically significant, with a chi-square value of 96.9285 and a p-value of 0.000 (Table 2).

**Table 2:** Educational status among urban and rural study participants.

Educational status	Rural n (%)	Urban n (%)
Illiterate	15 (7.1)	4 (1.9)
Class 1 – 12	127 (60.5)	38 (18.1)
Bachelor's degree	65 (31.0)	158 (75.2)
Master's degree	3 (1.4)	10 (4.8)
Total	210 (100)	210 (100)

In the rural group, 34.8% (n=73) were primigravida (first pregnancy), and 65.2% (n=137) were multigravida (multiple pregnancies). In the urban group, 38.1% (n=80) were primigravida and 61.9% (n=130) were multigravida. The difference in the distribution of obstetric scores between urban and rural participants was not statistically significant, with a chi-square value of 0.5038 and a p-value of 0.478 (Table 3) 

**Table 3:** Distribution of obstetric score among urban and rural study participants.

Obstetric score	Rural n (%)	Urban n (%)
Primigravida	73 (34.8)	80 (38.1)
Multigravida	137 (65.2)	130 (61.9)
Total	210 (100)	210 (100)

In the rural group, 38.6% (n=81) of participants had a normal vaginal delivery (NVD), and 61.4% (n=129) had a lower segment cesarean section (LSCS). In the urban group, 42.9% (n=90) of participants had a normal vaginal delivery, and 57.1% (n=120) had a cesarean section. The difference in the type of delivery between urban and rural participants was not statistically significant, with a chi-square value of 0.7990 and a p-value of 0.371 (Table 4).

**Table 4:** Distribution of type of delivery among urban and rural study participants. NVD: Normal vaginal delivery, LSCS: Lower segment cesarean section

Type of delivery	Rural n (%)	Urban n (%)
NVD	81 (38.6)	90 (42.9)
LSCS	129 (61.4)	120 (57.1)
Total	210 (100)	210 (100)

Urban mothers were significantly more likely than rural mothers to have received breastfeeding counseling during antenatal visits (56.7% vs. 41.4%) and were currently breastfeeding (83.8% vs. 73.8%) (Table 5).

**Table 5:** Comparison of responses to key questions on NICU admissions, breastfeeding, and formula feeding between urban and rural study participants. NICU: Neonatal intensive care unit, H/o: Head office

S.NO	Questions	Urban		Rural		p-value
		Yes n(%)	No n(%)	Yes n(%)	No n(%)	
1	H/o NICU admission for present baby	75 (35.7)	135 (64.3)	83 (39.5)	127 (60.5)	0.420
2	H/o NICU admissions for the previous baby (n=243)	36 (30.5)	82 (69.5)	26 (20.8)	99 (79.2)	0.083
3	H/o Death of the previous baby after breastfeeding initiation	18 (8.6)	192 (91.4)	12 (5.7)	198 (94.3)	0.256
4	Received counselling for breastfeeding during antenatal visits	119 (56.7)	91 (43.3)	87 (41.4)	123 (58.6)	0.002
5	Currently breastfeeding	176 (83.8)	34 (16.2)	155 (73.8)	55 (26.2)	0.012
6	Belief in formula feeding	96 (45.7)	114 (54.3)	109 (51.9)	101 (48.1)	0.204

Table 6 provides a detailed comparison of awareness, willingness, and beliefs about milk bank donation between Urban and rural participants, highlighting a significant disparity in prior knowledge (p= 0.016) and willingness to donate (p<0.001) (Table 6).

**Table 6:** Comparison of responses to key questions on awareness, willingness, and beliefs about milk bank donation between urban and rural study participants.

S.no	Questions	Urban		Rural		p-value
		Yes n(%)	No n(%)	Yes n(%)	No n(%)	
1	Previously heard about milk bank donations?	42 (20.0)	168 (80.0)	24 (11.4)	186 (88.6)	0.016
2	Do you feel very happy that my donated milk is the reason for the survival of a sick baby?	119 (56.7)	91 (43.3)	122 (58.1)	88 (41.9)	0.767
3	Is the mother willing to donate?	107 (50.9)	103 (49.1)	106 (50.5)	104 (49.5)	0.922
4	Is the mother willing to receive milk from the milk bank?	83 (39.5)	127 (60.5)	88 (41.9)	122 (58.1)	0.619
5	Requirement of spousal consent?	138 (65.7)	72 (34.3)	126 (60.0)	84 (40.0)	0.226
6	I believe that human milk donation can save babies.	126 (60.0)	84 (40.0)	126 (60.0)	84 (40.0)	1.000
7	I believe that human milk contains all the essential nutrients required for the healthy existence and complete development of the baby.	161 (76.7)	49 (23.3)	165 (78.6)	45 (21.4)	0.640
8	I believe that donating breast milk is good for a mother's health too as it reduces breast engorgement.	118 (56.2)	92 (43.8)	113 (53.8)	97 (46.2)	0.624
9	I believe that donating breast milk does not cause malnutrition in the mother or her baby.	111 (52.9)	99 (47.1)	102 (48.6)	108 (51.4)	0.380
10	I believe that there is nothing wrong with donating milk in case of excess production of milk.	120 (57.1)	90 (42.9)	118 (56.2)	92 (43.8)	0.844
11	I believe that my milk should be given only to my baby and not to others.	93 (44.3)	117 (55.7)	104 (49.5)	106 (50.5)	0.282
12	I am interested in donating milk but I am scared of expressing it using the machine	51 (45.1)	62 (54.9)	49 (43.8)	63 (56.3)	0.602
13	I believe the mother is willing to donate breast milk after the previous baby's NICU admission.	33 (97.1)	1 (2.9)	25 (89.3)	3 (10.7)	0.215
14	I believe that if I am giving my milk to other babies then it may not be sufficient for my baby.	105 (50.0)	105 (50.0)	104 (49.5)	106 (50.5)	0.922
15	I will donate milk only to my family members' and friend's babies and not to any strangers.	97 (46.2)	113 (53.8)	103 (49.1)	107 (51.0)	0.558

Urban mothers also had a higher awareness of milk bank donation (20.0% vs. 11.4%, p=0.016) (Table 7).

**Table 7:** Willingness to donate milk among study participants with and without previous knowledge.

S.NO	Questions	Willing to donate milk		p-value
		Yes n (%)	No n (%)	
1	Mothers with previous knowledge	57 (87.7)	8 (12.3)	<0.001
2	Mothers with no previous knowledge	156 (43.9)	199 (56.1)	

The logistic regression analysis showed that living in urban areas (AOR 2.01, 95% CI 1.30-2.96, p<0.001), having a higher level of education, prior knowledge of milk donation (AOR 7.56, 95% CI 3.73-15.8, p<0.001), and history of NICU admission for a previous baby (AOR 8.46, 95% CI 2.84-12.46, p<0.001) were significantly associated with an increased willingness to donate breast milk (Table 8). 

**Table 8:** Factors associated with willingness to donate milk among study participants. LSCS: Lower segment cesarean section, NVD: Normal vaginal delivery, NICU: Neonatal intensive care unit

Explanatory variable	Willingness to donate milk		COR (95%CI)	AOR (95% CI)	p-value
	Yes n (%)	No n (%)			
Age (in years)					
18 – 25	112 (45.9)	132 (54.1)	1	1	
26 – 36	101 (57.4)	75 (42.6)	1.59 (1.07 – 2.35)	1.52 (0.97 – 2.39)	0.069
Residence					
Rural	85 (40.5)	125 (59.5)	1	1	
Urban	128 (61.0)	82 (39.0)	2.29 (1.55 – 3.39)	2.01 (1.30 – 2.96)	<0.001
Educational status					
Illiterate	9 (47.4)	10 (52.6)	1	1	
Class 1 -12	82 (49.7)	83 (50.3)	2.06 (0.73 – 6.63)	1.37 (0.50 – 3.76)	0.546
Bachelor's degree	112 (50.2)	111 (49.8)	3.75 (1.34 – 11.98)	2.57 (1.11 – 8.42)	<0.001
Master's degree	10 (76.9)	3 (23.1)	8.58 (1.73 – 53.45)	6.13 (1.28 – 34.37)	<0.001
Monthly household income in a year					
0 -10000	31 (58.5)	22 (41.5)	1	1	
10001 – 20000	68 (45.6)	81 (54.4)	0.60 (0.32 – 1.12)	0.58 (0.29 – 1.15)	0.122
20001 - 30000	61 (46.9)	69 (53.1)	0.63 (0.33 – 1.20)	0.64 (0.27 – 1.48)	0.298
>30000	53 (60.0)	35 (40.0)	1.07 (0.54 – 2.15)	0.98 (0.40 – 2.42)	0.972
Religion					
Hindu	194 (51.2)	185 (48.8)	1	1	
Others	19 (46.3)	22 (53.7)	0.82 (0.43 – 1.57)	0.88 (0.43 – 1.77)	0.711
Gravida					
Primigravida	52 (34.0)	101 (66.0)	1	1	
Multigravida	161 (60.3)	106 (39.7)	2.94 (1.95 – 4.48)	2.65 (1.74 – 4.04)	<0.001
Type of delivery					
LSCS	92 (40.4)	136 (59.6)	1	1	<0.001
NVD	100 (58.5)	71 (41.5)	2.08 (1.39 – 3.12)	1.88 (1.15 – 2.88)	
No. of antenatal visit					
≤ 5	33 (60.0)	22 (40.0)	1	1	
> 5	180 (49.3)	185 (50.7)	0.65 (0.36 – 1.16)	0.77 (0.40 – 1.48)	0.430
Previously heard about milk donation					
No	156 (44.1)	198 (55.9)	1	1	
Yes	57 (86.4)	9 (13.6)	8.04 (3.86 – 16.7)	7.56 (3.73 – 15.8)	<0.001
Mothers with previous baby death					
No	186 (47.7)	204 (52.3)	1	1	
Yes	27 (90.0)	3 (10.0)	9.87 (2.95 – 33.07)	7.87 (2.12 – 24.76)	<0.001
Mothers with H/o NICU admission for previous baby					
No	69 (38.1)	112 (61.9)	1	1	
Yes	54 (87.1)	8 (12.9)	10.96 (4.92 – 24.40)	8.46 (2.84 – 12.46 )	<0.001
Mothers with H/o NICU admission for present baby					
No	114 (43.5)	148 (56.5)	1	1	
Yes	99 (62.7)	59 (37.3)	2.17 (1.45 – 3.26)	1.77 (1.15 – 2.96)	<0.001

## Discussion

This study investigated the impact of public health and mental health on mothers’ willingness to breastfeed and to donate human milk after childbirth in Vijayapura, with a comparison between urban and rural mothers. The findings reveal that a mother’s willingness to donate milk to milk banks is primarily influenced by their knowledge and understanding of the issue. The study identified significant variations in age, marital status, spouse’s income, and mother’s education level between urban and rural areas. These differences suggest that socio-economic factors may influence attitudes toward milk donation and should be considered when designing interventions. Urban women with higher education levels (bachelor’s or master’s degree), better family incomes, and those with multiple pregnancies or vaginal births (VVB) were more likely to prefer breastfeeding compared to rural mothers, reflecting a knowledge and education gap between the two groups.

The significant difference in donation rates between urban and rural mothers suggests that urban women have better access to resources, such as milk storage facilities and support networks. This disparity emphasizes the need for strategic interventions to support breastfeeding and educate about milk donation in rural areas. The study also revealed that prior information about breastfeeding increased mothers’ willingness to both donate and receive breast milk from breast milk banks. These results underscore the importance of widespread education campaigns to inform mothers about the benefits and safety of breastfeeding. Personal experiences, such as having a child previously hospitalized or experiencing the need for donor milk, also positively influence the mother's psychological willingness to donate. Mothers with firsthand experience of the crucial role breast milk plays in infant survival and health are more likely to donate. This suggests that sharing personal stories and testimonials from mothers who have benefited from breastfeeding and milk donation could serve as a powerful tool in encouraging others to participate in milk donation programs.

Additionally, the study found that 15.7% of women were knowledgeable about breast milk donation, while 50.7% expressed a desire to participate in milk donation. These findings are lower than Zhang N et al.'s (2019) Wuhan, China research, which found 20% had previous awareness and 81.3% were eager to give [22]. Both experiments show that previous knowledge increases donation. The Wuhan study's greater willingness rate shows cultural or environmental variables may be involved, highlighting a need for more focused teaching programs in Vijayapura. Sankar MN et al. (2019) found that parental knowledge had a significant impact on breastfeeding in the neonatal intensive care unit (NICU) which has a greater need for donations [23]. Our study revealed that moms having a history of NICU hospitalization for a previous or current infant were more likely to donate (AOR: 8.46, 1.77). These data show that personal newborn health issues boost empathy and milk donation support. Mondkar J et al.(2020) found that breastfeeding in India was hindered by a lack of knowledge and culture [24]. This suggests that education and high awareness of the importance of donation are important in increasing donations. Both studies addressed concerns about insufficient milk for their children.

They emphasized the need to improve education and environmental health to solve these problems. Monti L et al. (2023) found that altruism and healthcare professional support facilitated milk donation psychologically [25]. Our study also found that prior knowledge (AOR: 7.56) and good personal experiences, such as newborn NICU hospitalizations, motivate mothers to donate. This implies that promoting altruism and a friendly atmosphere might boost contribution rates. Although only 10% of respondents had little knowledge about breast milk banks, Verma A et al. (2016) showed high acceptance (85.4%) and donation (84.9%) in Bhopal, India [26]. In contrast, our study showed a moderate willingness to donate (50.7%) and accept (40.7%) breast milk, with 15.7% having prior knowledge. These differences underscore the need for awareness-raising initiatives in Vijayapura to bridge the gap between knowledge and interest. 

The significant association between higher education and willingness to donate suggests that educational interventions could be pivotal in increasing donation rates. Future research should focus on longitudinal studies to assess the long-term impact of these interventions on donation behavior. To increase confidence, education programs should prioritize breastfeeding benefits and safety measures. To encourage donation, hospital policies should provide widespread support for breastfeeding, especially for mothers in the neonatal intensive care unit (NICU). The use of social and behavioral change communication (SBCC) strategies during childcare can eliminate cultural and religious barriers to donation. Physicians should be trained to educate women about breastfeeding and to help them express and store breast milk. To gain public trust, authorities need to establish and maintain safe and effective breast milk banks. Finally, working with community and religious leaders can help eliminate negative attitudes and promote human milk donation, thereby helping to improve infant health. Ethical principles of respect for human dignity, beneficence, and justice should guide human milk donation to ensure all infants receive safe donor milk [27].

Despite its strengths, our study also has limitations that need to be acknowledged. The use of convenience sampling may have introduced selection bias, as participants were chosen based on their availability and willingness to participate, which could limit the representativeness of the sample. Additionally, the study was conducted in a specific geographic region, which may restrict the applicability of the findings to other settings with different cultural, social, or healthcare contexts.

## Conclusions

Our study revealed that urban residence, higher education levels, multi-parity, normal vaginal delivery, previous knowledge of milk donation, and having a child admitted to NICU are significant predictors of willingness to donate breast milk among lactating mothers in Vijayapura. These findings highlight that implementing targeted educational programs is crucial to enhancing milk donation rates, particularly in rural areas. Healthcare providers should be trained to counsel mothers on the benefits of milk donation while also explaining the safety protocols involved in donation and the safety of using donated milk. Public health campaigns should focus on increasing awareness and addressing cultural barriers. These insights can guide policymakers and healthcare providers in developing strategies to improve human milk banking practices and neonatal health outcomes.
